# Reliable perovskite indoor photovoltaics for self-powered devices

**DOI:** 10.1093/nsr/nwaf242

**Published:** 2025-06-12

**Authors:** Chun-Hao Chen, Xiao-Ying He, Rui-Hao Qin, Kai-Li Wang, Lei Huang, Run-Jun Jin, Xin Chen, Ze-Kai Bian, Yu-Tong Yang, Kai Jin, Jing Chen, Yu Xia, Ilhan Yavuz, Zhao-Kui Wang

**Affiliations:** State Key Laboratory of Bioinspired Interfacial Materials Science, Institute of Functional Nano & Soft Materials (FUNSOM), Soochow University, Suzhou 215123, China; State Key Laboratory of Bioinspired Interfacial Materials Science, Institute of Functional Nano & Soft Materials (FUNSOM), Soochow University, Suzhou 215123, China; State Key Laboratory of Bioinspired Interfacial Materials Science, Institute of Functional Nano & Soft Materials (FUNSOM), Soochow University, Suzhou 215123, China; State Key Laboratory of Bioinspired Interfacial Materials Science, Institute of Functional Nano & Soft Materials (FUNSOM), Soochow University, Suzhou 215123, China; State Key Laboratory of Bioinspired Interfacial Materials Science, Institute of Functional Nano & Soft Materials (FUNSOM), Soochow University, Suzhou 215123, China; State Key Laboratory of Bioinspired Interfacial Materials Science, Institute of Functional Nano & Soft Materials (FUNSOM), Soochow University, Suzhou 215123, China; State Key Laboratory of Bioinspired Interfacial Materials Science, Institute of Functional Nano & Soft Materials (FUNSOM), Soochow University, Suzhou 215123, China; State Key Laboratory of Bioinspired Interfacial Materials Science, Institute of Functional Nano & Soft Materials (FUNSOM), Soochow University, Suzhou 215123, China; State Key Laboratory of Bioinspired Interfacial Materials Science, Institute of Functional Nano & Soft Materials (FUNSOM), Soochow University, Suzhou 215123, China; State Key Laboratory of Bioinspired Interfacial Materials Science, Institute of Functional Nano & Soft Materials (FUNSOM), Soochow University, Suzhou 215123, China; State Key Laboratory of Bioinspired Interfacial Materials Science, Institute of Functional Nano & Soft Materials (FUNSOM), Soochow University, Suzhou 215123, China; State Key Laboratory of Bioinspired Interfacial Materials Science, Institute of Functional Nano & Soft Materials (FUNSOM), Soochow University, Suzhou 215123, China; Department of Physics, Marmara University, Ziverbey 34722, Türkiye; State Key Laboratory of Bioinspired Interfacial Materials Science, Institute of Functional Nano & Soft Materials (FUNSOM), Soochow University, Suzhou 215123, China

**Keywords:** perovskite indoor photovoltaics, Internet of Things, self-assembled monolayer, device stability

## Abstract

The rise of the Internet of Things has spurred a rapid increase in demand for self-powered devices, accompanied by the swift development of highly compatible indoor photovoltaics. Perovskite indoor photovoltaics (PIPVs) have garnered significant attention due to their tunable bandgaps, high output voltage etc. Long-term stability is the most critical hurdle for the commercialization of PIPVs and is under intense market scrutiny. The stability of self-assembled monolayers in inverted PIPVs plays a pivotal role in determining device stability. In this work, we employ an interlocking self-assembled monolayer strategy that enhances device stability by improving film coverage. Optimized devices achieve a record indoor photovoltaic power conversion efficiency of 42.01% under 1000 lx illumination. Furthermore, in the accelerated aging stability test simulating a day/night cycle with light intensity fluctuating from 2000 to 0 lx, the projected T90 lifespan of the final target device approaches 6000 h. Ultimately, the PIPV module, through the integration of integrated circuits, achieved continuous and reliable operation of an electronic price tag.

## Teaser

This work develops reliable perovskite indoor photovoltaics for IoT devices, achieving record 42% efficiency and projected ∼6000-hour lifespan under indoor light, enabling continuous power for micro-energy electronics.

## INTRODUCTION

The Internet of Things (IoT) envisions the interconnection of all uniquely addressable physical objects, facilitating a networked world where devices can communicate and interact autonomously [1,2]. Since its inception in the late 20th century, IoT has experienced rapid advancements, resulting in a proliferation of self-powered devices such as sensors, terminal units and health monitors within these systems. This expansion has highlighted an increasingly apparent gap in energy demand. Given that IoT systems operate via wireless networks, the integration of off-grid power solutions enhances system stability and independence. Therefore, the development of an efficient, stable and self-powered technology capable of harvesting energy directly from the surrounding environment has become imperative [3]. Indoor photovoltaics (IPVs) have emerged as a critical energy support technology for IoT, as they can effectively address these stringent criteria [4,5]. Various photovoltaic technologies, including amorphous silicon (a-Si), [6] dye-sensitized solar cells (DSSCs) [7,8], organic photovoltaics (OPVs) [9,10] and perovskite solar cells (PSCs) [11,12], have proved to be suitable for IPV applications. Among these, perovskite indoor photovoltaics (PIPVs) stand out due to their tunable bandgap, high theoretical efficiency, flexible fabrication capabilities and low cost [13,14].

Compared to large-scale solar power stations, PIPVs benefit from smaller deployment volumes and milder operating environments, which facilitate a smoother path to commercialization [15,16]. Nevertheless, several critical challenges must be addressed before PIPVs can fully transition to the market: ensuring that operational lifespans meet market expectations [17–19]; accommodating customized device requirements (such as varying shapes and flexible devices) [20–22]; and meeting specified performance thresholds for output voltage, current or power [23–25]. While significant advancements have been made in the development of flexible devices and the optimization of output parameters, comprehensive studies addressing the longevity of PIPVs remain limited. This study focuses on the stability of PIPVs, conducting a series of accelerated aging tests to assess whether their operational lifespans meet market requirements [26–28].

Here, we employed an interlocking self-assembled monolayer (SAM) strategy, substituting half of the typical SAM material, [4-(7H-dibenzo[c,g]carbazol-7-yl)butyl]phosphonic acid (4PADCB)] [29,30], with [4-(7H-dibenzo[c,g]carbazol-7-yl)ethyl]phosphonic acid (2PADCB). The combination of SAM materials with varying carbon chain lengths effectively increased surface coverage on indium tin oxide (ITO) and enhanced the binding energy between the SAM and ITO. Consequently, thermal stability was significantly improved, allowing target devices to perform better under higher annealing temperatures and during 85°C thermal stability tests. The optimized devices achieved an indoor power conversion efficiency (IPCE) of 42.01% under 1000 lx [light-emitting diode (LED), 3000 K], with an open-circuit voltage (*V*_OC_) of 1.07 V and fill factor (FF) of 84.22%, setting a record for PIPV efficiency. Moreover, we performed accelerated stability tests simulating diurnal cycles, subjecting the devices to alternating light/dark conditions (2000 to 0 lx). This protocol expedited the aging process, allowing us to estimate a T90 lifetime of nearly 6000 h for the target devices. To accelerate the commercialization of PIPVs, we developed an initial prototype that integrates PIPVs with self-powered devices. Our research demonstrates the practical feasibility of PIPVs in real-world applications, showing promising long-term performance for off-grid solutions. This study proposes stability optimization strategies and establishes a systematic evaluation methodology for PIPVs. The integrated system combining PIPV technology with self-powered devices makes a significant contribution to advancing their commercial applications.

## RESULTS AND DISCUSSION

### Thermal stability of interlocking SAMs

**Figure 1.**
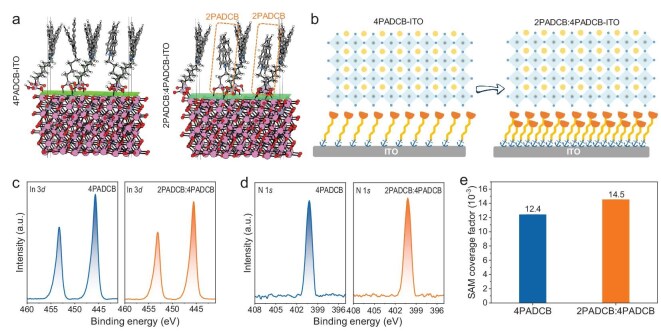
The morphology of interlocking SAM films. (a) The side-view image of theoretical SAM morphology on ITO. (b) Schematic diagram of 4PADCB and interlocking SAM morphology. (c) XPS of indium 3*d* core-level spectra on the SAM films. (d) XPS of N 1*s* core-level spectra on the SAM films. (e) The coverage factor of SAMs (4PADCB and 2PADCB:4PADCB = 1:1) on ITO.

Initially, we investigated the effects of substituting 4PADCB with 2PADCB at varying ratios (the structural diagrams of both SAM molecules are shown in Fig. S1 in the online supplementary file). Our findings revealed that a 1:1 ratio of 2PADCB to 4PADCB yields optimal indoor performance for perovskite photovoltaic devices (Fig. S2). Specifically, when pure 4PADCB was employed as a SAM layer, the device achieved an optimal IPCE of 39.76%. Utilizing pure 2PADCB for the SAM layer resulted in an optimal IPCE of 37.17%. Notably, a 1:1 mixture of 2PADCB and 4PADCB led to a significant enhancement, with the optimal IPCE reaching 42.01%. We performed further morphological analysis on both the pristine (pure 4PADCB on ITO) and target (2PADCB:4PADCB 1:1 blend) samples using density functional theory (DFT) calculations, examining side-view and top-view images (Fig. 1a, Fig. S3). The differing carbon chain lengths allowed 2PADCB to more readily fill gaps within the 4PADCB matrix with an interlocking structure, leading to an average intermolecular interaction energy of −0.33 eV for the 2PADCB:4PADCB (1:1) mixture, significantly higher than the −0.19 eV observed for standalone 4PADCB (Fig. S4). As schematically shown in Fig. 1b, this approach achieved greater surface coverage compared to the 4PADCB SAM layer, which is beneficial for subsequent crystallization growth of the perovskite layer. The X-ray Diffraction (XRD) results demonstrated improved crystallization of the perovskite film deposited on the mixed SAM (Fig. S5). The interlocking SAM exhibits superior wetting characteristics towards the perovskite precursor solution, as evidenced by the smaller contact angles observed (Fig. S6). The contact angles for the perovskite solution on 4PADCB and the interlocking SAM are 34.9° and 30.1°, respectively. This enhanced wetting facilitates the deposition of the perovskite film and promotes high-quality crystallization. We investigated the surface roughness of the SAM films using atomic force microscopy (AFM), and the results indicated that the compact arrangement in the interlocking SAM led to a reduction in surface roughness (Fig. S7). Through the application of X-ray photoelectron spectroscopy (XPS), we quantified the coverage factor of the two kinds of SAMs on ITO (Fig. 1c–e). The coverage factor, a relative measure of SAM molecular coverage, was determined by normalizing the nitrogen 1*s* core level area to the indium 3*d*_3/2_ core level area. When 2PADCB and 4PADCB were mixed at a 1:1 ratio, the coverage factor on ITO reached 14.5 × 10^−3^, surpassing the 12.4 × 10^−3^ for 4PADCB alone, thereby confirming the effectiveness of the interlocking SAM strategy in enhancing surface coverage density. Kelvin probe force microscopy (KPFM) measurements were conducted to assess the uniformity of the SAM layer on the ITO surface (Fig. S8). Corresponding contact potential difference (CPD) maps were simultaneously obtained and plotted for each sample (Fig. S9). Compared to the 4PADCB sample, the 2PADCB:4PADCB sample exhibited a relatively more uniform CPD (from ∼35 to ∼30 mV), which can be attributed to the effectiveness of our interlocking SAM strategy.

**Figure 2.**
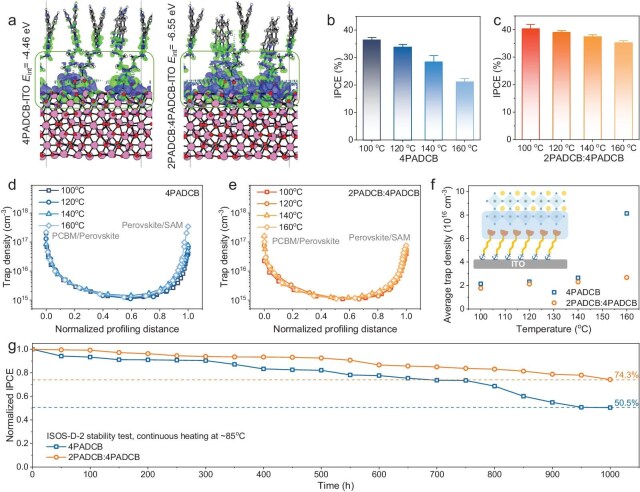
The thermal stability of PIPVs with interlocking SAMs. (a) Theoretical models of the interaction between SAMs and ITO. (b) The IPCE distribution of the 4PADCB-based PIPVs under different annealing temperatures. (c) The IPCE distribution of the interlocking SAM-based PIPVs under different annealing temperatures. (d) DLCP curves of the 4PADCB-based PIPVs under different annealing temperatures. (e) DLCP curves of the interlocking SAM-based PIPVs under different annealing temperatures. (f) The average trap density of the buried interface of perovskite films. (g) ISOS-D-2 stability test of the pristine and target PIPVs.

The binding energy between the two types of SAM layer and ITO was also evaluated by DFT calculation (Fig. 2a). The DFT-calculated charge-density difference (CDD) results in Fig. 2a reveal a strong charge accumulation at the 2PADCB:4PADCB–ITO interface compared to 4PADCB–ITO, pointing to the strong interaction of the former. The interaction energy for the interlocking SAM with ITO was −6.55 eV, stronger than the −4.46 eV for 4PADCB. This enhanced anchoring strength reduces the likelihood of SAM desorption, thereby contributing to improved thermal stability of the perovskite photovoltaic devices. Annealing the SAM films for 10 min at different temperatures demonstrated that devices incorporating 4PADCB showed a marked decrease in efficiency. At 100°C, these devices averaged an IPCE of 36.37%, which declined to 21.23% at 160°C, marking a substantial decrease of 41.6% (Fig. 2b). This temperature-dependent efficiency loss underscores the thermal sensitivity of 4PADCB-based devices. Conversely, devices fabricated with the 2PADCB:4PADCB (1:1) mixture demonstrated a significantly more stable performance under thermal stress. The IPCE reduction was limited to 12.6%, declining from 40.38% at 100°C to 35.28% at 160°C (Fig. 2c). This comparatively minor decrease highlights the superior thermal stability of the interlocking SAM in perovskite photovoltaic devices. This superior heat tolerance of the interlocking SAM over pure 4PADCB can be attributed to reduced desorption and disorder at higher annealing temperatures, which would otherwise increase the trap density of states at the SAM/perovskite interface. To evaluate the trap density of states, deep-level capacitance profiling (DLCP) curves were measured under different annealing temperatures (Fig. 2d and e). Both sets of devices exhibited increased trap densities at the buried perovskite interface as annealing temperature rose from 100°C to 160°C. We calculated the average trap density for the bottom 10% thickness of the perovskite layer (Fig. 2f). For 4PADCB-based devices, the average trap density rose from 2.14 × 10^16^ to 8.13 × 10^16^ cm^−3^, representing a significant 280% increase. In contrast, whereas for the interlocking SAM-based devices, the average trap density increased from 1.76 × 10^16^ to 2.68 × 10^16^ cm^−3^, corresponding to a more modest 52% increase. These findings further underscore the enhanced thermal stability exhibited by the interlocking SAM layer, demonstrating its effectiveness in maintaining performance under elevated temperatures. Importantly, we conducted storage stability tests according to the International Summit on Organic Photovoltaic Stability (ISOS)-D-2 protocol, subjecting the samples to continuous heating at 85°C for an extended period (Fig. 2g). After 1000 h, the IPCE of the pristine device dropped to 50.5% of its initial value, whereas that of the target device retained 74.3% of its initial performance. The target device demonstrated significantly improved thermal stability, highlighting the benefits of the interlocking SAM strategy in advancing PIPV technology towards commercial viability.

### Indoor photovoltaic performance

**Figure 3.**
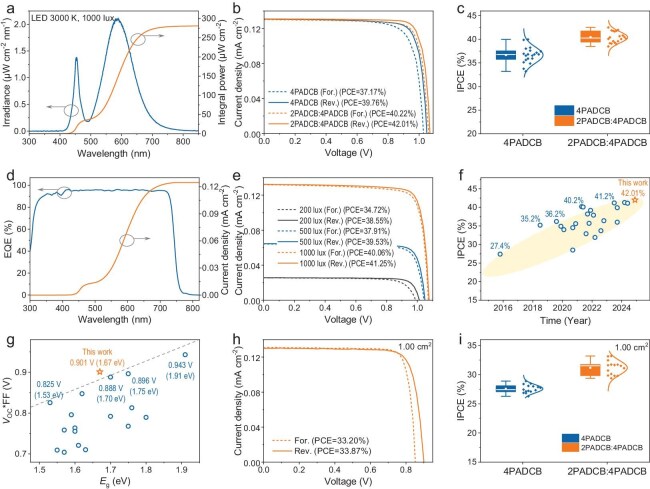
Indoor photovoltaic performance. (a) The spectrum of LED 3000 K light source under 1000 lx illuminance and the corresponding integrated light source power. (b) *J-V* curves of pristine and target devices in the reverse and forward scans under 1000 lx. (c) The IPCE distribution of devices for pristine and target PIPVs. (d) The EQE spectrum and the integrated current density of the target devices. (e) *J-V* curves measured under 200, 500 and 1000 lx of the target PIPVs in the reverse and forward scans. (f) Summary of IPCEs of the published PIPVs in recent years. (g) Summary of *V*_OC_*FF of the published PIPVs in recent years. (h) *J-V* curves of target devices (1 cm^2^) in the reverse and forward scans under 1000 lx. (i) The IPCE distribution of devices (1 cm^2^) for pristine and target PIPVs.

To evaluate the indoor performance of the devices, we utilized LED lighting with a color temperature of 3000 K, adjusted to an illuminance level of 1000 lx, mimicking typical indoor conditions. The indoor light spectrum was characterized (Fig. 3a), and its power density, calculated by integrating the spectral irradiance, was determined to be 280 μW/cm^2^. To optimize device performance under this specific spectrum, we fine-tuned the perovskite bandgap and composition. Detailed fabrication methods are provided in the Supporting Information. UV-vis absorption spectra confirmed a consistent perovskite bandgap of 1.67 eV across different substrates (Fig. S10). This approach ensures that the perovskite photovoltaic devices are finely tuned to indoor lighting conditions, achieving superior performance and reliability. By precisely matching the material properties to the operating environment, we have developed high-performance photovoltaic devices tailored for indoor applications.

Under 1000 lx illumination, the IPCEs of the two types of devices were measured as follows: for the pristine device, an IPCE of 39.76% [*V*_OC_ = 1.05 V, short-circuit current density (*J*_SC_) = 0.131 mA/cm^2^, FF = 81.38%]; and for the target device, an efficiency of 42.01% (*V*_OC_ = 1.07 V, *J*_SC_ = 0.131 mA/cm^2^, FF = 84.22%) using the reverse scan measurement method (Fig. 3b and Table S1). The improved performance of the target device is attributed to better contact at the SAM/perovskite interface and higher-quality SAM film, which significantly enhanced both *V*_OC_ and FF. Additionally, the parameters of the *J-V* curves by the forward scan mode listed in Table S1 show reduced hysteresis in the target device. Figure 3c presents the IPCE distribution of 20 devices under 1000 lx, showing a Gaussian distribution for both pristine and target samples. The average IPCE of the target PIPVs was approximately 10.3% higher than that of the original sample (from 36.66% to 40.42%). External quantum efficiency (EQE) measurements (Fig. 3d) indicated an integrated current density of 0.125 mA/cm^2^ under the spectrum shown in Fig. 3a, aligning well with actual current densities.

We further investigated the IPCE of our devices under varying illumination intensities corresponding to input power densities of 285 (1000 lx), 147 (500 lx) and 55 μW/cm^2^ (200 lx) (Fig. 3e, Fig. S11). For a comprehensive overview, the detailed IPCE parameters measured under different light intensities are summarized in Table S2. At an illumination level of 1000 lx, the output power density of the PIPV reached an impressive 118 μW/cm^2^. The consistent high efficiency at various illuminance levels demonstrates the reliability of the devices. Moreover, the enhanced *V*_OC_ and FF indicate that our devices are better optimized for energy harvesting under low-light conditions, a critical factor for indoor photovoltaic technology. Notably, our highest IPCE exceeds recent reports on PIPVs (Fig. 3f, Table S3). Additionally, we have summarized the perovskite bandgap (*E*_g_) and the *V*_OC_*FF parameter from recent work on PIPVs (under illumination levels of approximately 1000 lx). In our work, the *V*_OC_*FF parameter significantly outperforms other reported values for devices with similar perovskite bandgaps (Fig. 3g, Table S3). This highlights the superior selection of the perovskite bandgap in our study and the excellence of the fabrication techniques employed.

Furthermore, we fabricated large-area devices with an active area of 1.00 cm^2^, achieving an impressive indoor efficiency of 33.87% under reverse scans (Fig. 3h, detailed parameters are provided in Table S1). Figure 3i illustrates the IPCE distribution of these 1.00 cm^2^ devices under 1000 lx illumination, demonstrating a notable increase in average IPCE from 27.57% for the original sample to 31.18% for the optimized device. The enhanced performance of the large-area device highlights the effectiveness of the optimization strategies. The consistent improvement in IPCE across multiple devices underscores the reproducibility of our fabrication process. The comprehensive characterization and performance data of these large-area devices offer critical evidence that supports the potential for further application and commercialization of PIPVs.

**Figure 4.**
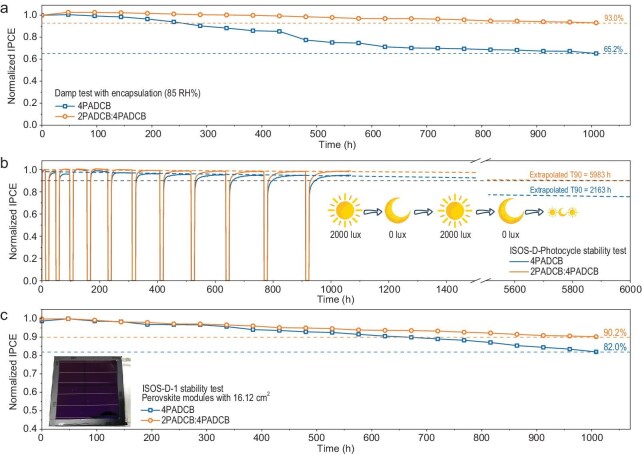
Damp and ISOS-D tests of PIPVs. (a) Damp stability test of the pristine and target PIPVs. (b) ISOS-D-Photocycle stability test of the pristine and target PIPVs. (c) ISOS-D-1 stability test of the pristine and target PIPV modules (16.12 cm^2^).

To validate the impact of an interlocking SAM on device stability, we conducted an extensive series of accelerated aging tests. For humidity stability assessments, encapsulated devices were exposed to 85% relative humidity over extended periods. After 1000 h of this rigorous accelerated aging, the indoor efficiency of the reference device deteriorated to only 65.2% of its initial value, whereas the IPCE of the device optimized with an interlocking SAM remained at 93.0% (Fig. 4a). The enhanced moisture stability of the optimized devices can be attributed to the increased binding energy of the interlocking SAM molecules and their improved binding energy with the ITO surface. The degradation mechanisms of PIPVs often exhibit full or partial reversibility in the dark, a phenomenon known as metastability. To better understand device behavior under realistic diurnal cycles, we employed the ISOS-D-Photocycle protocol, which alternates between light and dark phases. This cycling presents a different stress scenario compared to continuous illumination (ISOS-L protocol) and more accurately simulates real-world conditions. PIPVs were exposed to 2000 lx illumination turned on and off with cycle periods of 12 h and duty cycles (light to dark) of n:1, where n is the time of switch-off before this switch-on operation. Of the suggested conditions, 12(n + 1)-h-long cycles (12 h light and 12 h dark, 24 h light and 12 h dark, 36 h light and 12 h dark) mimic the diurnal cycle. Because the interplay between degradation and recovery in realistic conditions can be complex and depend on cell history, varying cycle duration and duty cycle should provide additional information on the extent of reversibility and sufficient recovery times. Detailed testing methods are described in Supplementary Note S3. The device incorporating an interlocking SAM maintained over 98.5% of its maximum IPCE after 12 light/dark cycles. Linear extrapolation indicated an impressive T90 lifetime of 5983 h. The projected lifetime is based on linear extrapolation, while actual aging processes may exhibit non-linear degradation behavior, potentially resulting in lower stability than estimated. [27] In stark contrast, the T90 lifetime for the 4PADCB-based device was significantly shorter, at 2163 h (Fig. 4b). We also fabricated large-area modules with an effective area of 16.12 cm^2^ (Fig. 4c) to evaluate the scalability of our approach. Under the ISOS-D-1 stability protocol, these encapsulated modules retained 90.2% (for the target devices) and 82.0% (for the pristine devices) of their maximum IPCE after 1000 h, respectively. These results demonstrate that our strategy remains effective even when scaled up to larger formats, ensuring consistent performance across different device sizes.

### PIPV self-powered devices

**Figure 5.**
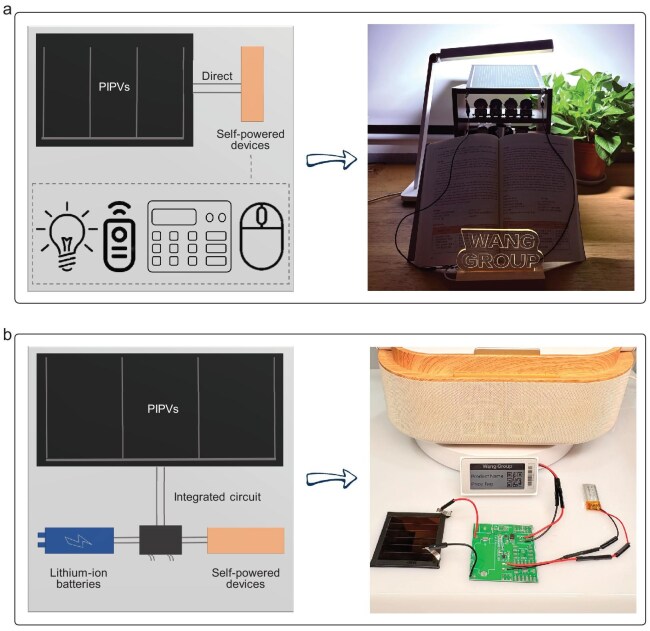
Actual circuit diagram and self-powered applications of PIPVs. (a) Schematic diagram of direct connection circuit for PIPVs with self-powered devices. The driving devices can be LED, remote control, calculator and mouse etc. The right photo shows the yellow LED driven by PIPV modules. (b) The integrated circuit diagram, including integrated circuit boards, PIPVs, lithium batteries and self-powered devices such as electronic price tags. The right photo shows electronic price tags driven by the integrated circuit diagram.

To facilitate the commercialization of PIPVs, we have developed an initial prototype of self-powered devices. Figure 5a illustrates the schematic diagram of the direct connection circuit for PIPVs integrated with self-powered devices. Typical self-powered devices include LEDs, remote controls, calculators, mice and electronic price tags. We successfully demonstrated the operation of a yellow LED driven by a PIPV module under desk-lamp illumination using a simple direct connection circuit. However, the continuous power supply requirement for indoor light sources is stringent; the device ceases to function when placed in darkness. Therefore, integrating energy storage devices such as lithium-ion batteries is essential. Figure 5b presents the integrated circuit diagram, which includes the integrated circuit board, PIPV, lithium-ion battery and self-powered device (Fig. S12). Through this integrated circuit, we achieved a continuous power supply to electronic price tags. The voltage requirements for this type of electronic price tag range from 2.4 to 3.6 V. Due to the excellent voltage output performance of PIPVs under indoor lighting conditions, a series connection of about three sub-cells can meet the voltage requirements of the electronic price tags. Different self-powered devices have varying demands on the output parameters of PIPVs (e.g. power or current output requirements), necessitating tailored design and adjustment of PIPVs for different products.

After meeting the fundamental driving requirements, the lifespan of PIPVs was estimated. Further linear extrapolation predictions for the lifetime of ISOS-D-Photocycle devices suggest a T50 of about 3.5 years. More advanced encapsulation of PIPVs and better operational environment designs will further enhance the operational lifespan of PIPVs, thereby preliminarily satisfying the basic lifespan requirements for commercialization.

## CONCLUSION

We significantly advance the commercial viability of PIPVs for IoT applications. By employing an interlocking SAM strategy, we enhanced device thermal stability and achieved superior photovoltaic performance, including a record indoor efficiency of 42.01% under 1000 lx. Accelerated aging tests under simulated diurnal cycles indicated a promising T90 lifetime nearing 6000 h, addressing critical lifespan concerns. These improvements in efficiency and stability are crucial milestones towards meeting market expectations for off-grid power solutions. Furthermore, the successful integration of PIPVs with self-powered devices demonstrates their practical applicability in real-world scenarios. The systematic research approach adopted here provides a robust framework for future studies, facilitating the transition of PIPVs from laboratory achievements to commercially viable products.

## Supplementary Material

nwaf242_Supplemental_File
