# Supplementary material for: Reliable perovskite indoor photovoltaics for self-powered devices
Source: Natl Sci Rev. 2025 Jun 12;12(8):nwaf242. doi: 10.1093/nsr/nwaf242 (PMC12236333; doi:10.1093/nsr/nwaf242)
Supplement: nwaf242_Supplemental_File [file nwaf242_supplemental_file.pdf]

Supplementary Materials For

Reliable perovskite indoor photovoltaics for self-powered devices

Chun-Hao Chen[1,†], Xiao-Ying He[1,†], Rui-Hao Qin[1,†], Kai-Li Wang[1], Lei Huang[1], Run-Jun Jin[1], Xin Chen[1], Ze-Kai Bian[1], Yu-Tong Yang[1], Kai Jin[1], Jing Chen[1], Yu Xia[1], Ilhan Yavuz[2] and Zhao-Kui Wang[1,*]

[1]State Key Laboratory of Bioinspired Interfacial Materials Science, Institute of Functional Nano & Soft Materials (FUNSOM), Soochow University, Suzhou 215123, China; [2]Department of Physics, Marmara University, Ziverbey 34722, Türkiye

*__Corresponding author__. E-mail: zkwang@suda.edu.cn

[†]Equally contributed to this work.

This PDF file includes:

Materials and Methods

Supplementary Note S1-S4

Figures S1-S12

Tables S1-S3

References (*1*-*28*)

Materials

Lead (II) bromide (99.99%) were purchased from Sigma-Aldrich. CsI (99.99%) were purchased from Yingkou Libra New Energy Technology Co., Ltd. MACl (99.99%), FAI (99.99%) and MABr (99.99%) were purchased from Greatcell Solar. DMF (>99.8%), DMSO (>99.8%), chlorobenzene (CB, >99.8%), IPA and ethanol (EtOH, >99.8%) were purchased from Sigma-Aldrich. PCBM was purchased from Funano Co.,Ltd. 2PADCB (98%), 4PADCB (98%) and C60 (99%) was purchased from Xi'an Yuri Solar Co., Ltd. Pre-patterned ITO glass, Lead (II) iodide (99.999%), Bathocuproine (BCP) were obtained from Advanced Election Technology Co., Ltd. All the chemicals were used as received without purification.

SAM solution was made for 1 mmol L^{-1}. The mixed SAM solution w as prepared by first dissolving each component separately according to the desired molar ratio, followed by combining the individual solutions to obtain the final mixture. For $Cs_{0.05}(FA_{0.75}MA_{0.25})_{0.95}Pb(I_{0.75}Br_{0.25})_3$-based solar cells, a 1.4 mol L^{-1} solution was prepared in mixed solvents of DMF and DMSO (v/v = 8:1). 20 mg PCBM was dissolved in 1 mL CB. The solutions were stirred overnight before usage.

Method

Device Fabrication.

The PSCs were fabricated with a structure of ITO/SAM/Perovskite/PCBM/BCP/Ag. The ITO-coated glass substrates underwent a cleaning protocol involving detergent and 15-minute rinses in deionized water, acetone, and ethanol. After being dried in an oven for over 3 hours, they were treated with ultraviolet ozone for 20 minutes. The SAM layer was deposited by spin-coating at 3000 rpm for 40 seconds and then annealed at 100 ℃ for 10 minutes. Perovskite films were fabricated through a two-step spin-coating process, conducted at 1000 rpm for 10 seconds and 6000 rpm for 30 seconds, with the addition of CB anti-solvent at 25 seconds, and subsequently annealed at 100 ℃ for 30 minutes. PCBM layer was deposited at 2000 rpm for 30 seconds. The substrates were then introduced into a thermal evaporation chamber, where C_{60} (30 nm), BCP (8 nm) and Ag (80 nm) layers were deposited in sequence via vacuum evaporation at a pressure of 3×10^{-4} Pa, with respective deposition rates of 0.5, 0.2 and 2 Å s^{-1}. The effective area of the resulting device was 1 cm^2 and 0.0669 cm^2.

For the Mini module, a 5×5 cm^2 large-area perovskite module was etched using processes P1, P2, and P3. First, the substrate was ultrasonically cleaned with acetone and ethanol, each for 15 minutes. The P1 scribe line width was 20-30 μm, and laser ablation was employed with relatively high laser power to ensure effective etching of the ITO substrate. The fabrication methods for each functional layer of the device were the same as those used for small-size PSCs. After deposition of the electron transport layer, P2 laser scribing was carried out with a line width of 180-200 μm, also using laser ablation, and the etching was terminated at the ITO substrate. Subsequently, a 100 nm-thick silver electrode was thermally evaporated. The P3 scribe line width was 60-80 μm, which was also completed via laser ablation, terminating the etching at the ITO substrate.

Characterization. XPS spectra was acquired using a Kratos AXIS Ultra-DLD ultrahigh vacuum surface analysis system. The films' morphology and electronic structure were

characterized by AFM using a Bruker Dimensional Icon with MESP-RC probes. PL spectra were measured at an excitation wavelength of 365 nm using a Horiba Jobin-Yvon Lab RAM HR800. A Keithley 2400 source meter was used to measure the *J*-*V* characteristics of PSCs (0.0669 cm^2 and 1 cm^2) under 1000 lux, 500 lux and 200 lux. Operational stability studies were conducted according to the ISOS-D-1 protocol, thinner masks were used, and anti-reflection treatment was applied. For EQE measurements, a system including a xenon lamp, monochromator, chopper, lock-in amplifier, and a calibrated silicon photodetector was employed. No pre-conditioning, such as light-soaking or bias voltage, was applied to any devices. The IM6e Electrochemical Workstation (ZAHNER, Germany) facilitated standard *C*-*V*, DLCP, and TAS measurements, while *C*-*f* analysis was carried out with an Agilent 4294A precision impedance analyzer.

Supplementary Note S1.

DFT calculations.

We employ the VASP program for plane-wave Density Functional Theory (DFT).[1,2] In our calculations, we used an energy cut-off of 300 eV for the plane-wave basis set. We choose a PBEsol to describe the exchange-correlation energies and pseudo-potential functions to represent core electrons using the standard projector-augmented wave (PAW) method. For the self-consistent field energy calculations, an energy threshold of 10^{-4} eV is chosen. The geometries are allowed to relax until all residual forces on ions are below 0.02 eV/Å. We choose a minimum of 2x2x1 grid for k-points.

Interaction energy between the SAM and ITO and between the SAM molecules is calculated by $\Delta E_{int} = E_{AB} - (E_A + E_B)$.

CDD analysis. We analyzed the tendency of charge transfer and interface interaction using the charge density difference (CDD), $\Delta\rho = \rho_{ITO+SAM} - (\rho_{ITO} + \rho_{SAM})$. The increase of $\Delta\rho$ in positive or negative direction means charge delocalization, hence, the increase in charge transfer. $\Delta\rho < 0$ means local electron depletion, $\Delta\rho > 0$ means local electron increase.

Supplementary Note S2.

Encapsulation and stability measurements.

The devices were encapsulated in a nitrogen glovebox. Both individual cells and modules were encapsulated using a combination of edge-sealing with UV-curable epoxy (EpoTech, OG159-2) and blanket-sealing with polyolefin encapsulant (Mitsui Solar TR02BA). To assess the heat performance of the encapsulated devices, the solar cells were exposed to a constant temperature and humidity of 85 ℃ and 25% R.H. in the testing chamber. Measurements were taken periodically after the devices had cooled. For humidity stability studies, operational stability studies were conducted according to the ISOS-D-2 protocol while the devices were put in a testing chamber with controlled humidity of 85% R.H. and temperature of 25 ℃. The current density versus voltage (*J*-*V*) characteristics were measured regularly under LED indoor light condition (@1000 lux) by using a programmable Keithley 2400 source meter. The indoor light system and HS-IL spectrometer were provided from enlitech. All the devices were measured without pre-conditioning such as light-soaking and applied bias voltage. Typically, the *J*-*V* curves of devices were measured in reverse scan (1.3 →-0.2 V, step 0.02 V, delay time 10 ms), and forward scan (-0.2→1.3 V, step 0.02 V, delay time 10 ms). After each test, the devices were immediately placed in the test chamber until the next test.

Supplementary Note S3.

ISOS-D-Photocycle stability measurements.

PSC degradation mechanisms have frequently demonstrated full or partial reversibility in dark conditions (a phenomenon often termed metastability). As a result, cycling between light and dark phases to mimic day-night transitions presents a markedly different stress scenario compared to continuous illumination (as per ISOS-L protocols). Literature reports highlight two contrasting dynamic behaviors: a reversible light-induced increase in PCE followed by a reduction in the dark, and photo-induced degradation with recovery in the dark.

PIPVs were exposed to ~2000 lux illumination turned on and off with cycle periods of 12 h and duty cycles (light:dark) of n:1, where n is the times of switch off before this switch on operation. Of the suggested conditions, 12(n+1)-h-long cycles (12 h light and 12 h dark, 24 h light and 12 h dark, 36 h light and 12 h dark) mimic the diurnal cycle. However, because the interplay between degradation and recovery in realistic conditions can be complex and depend on cell history, varying cycle duration and duty cycle should provide additional information on the extent of reversibility and sufficient recovery times.

The ISOS-D-Photocycle Stability Test was performed by using thin film photovoltaic test system (Fluxim AG, Litos Lite) and samples were not encapsulated. The test data was collected every 60 minutes with periodically switched on/off (or day-night) light source. The periodically switched light source was realized by code. Switch off time is 12 h while switch on time is 12(n+1), where n is the times of switch off before this switch on operation, under room temperature with nitrogen atmosphere. The load is the MPP point voltage and the open voltage.

Supplementary Note S4.

DLCP measurement: The DLCP method uses a variable δV (e.g., 20 to 200 mV) to measure the junction capacitance and acquire the capacitance contribution from the trap states by taking advantage of the information embedded in the higher order terms. With the determination of C_0 and C_1, the carrier density (N) that includes both free carrier density (N_0) and trap density (N_T) at a certain position X from the junction barrier was calculated by:[3]

$$N = -\frac{C_0^3}{2q\varepsilon A^2 C_1} \quad \text{Eq. S1}$$

where ε=6.5 is the dielectric constant of perovskite, q is the elementary charge, A=0.0669 cm^2 is the active area of the cells. The trap density of samples was estimated by subtracting the estimated free carrier density. The free carrier density is estimated by measuring the carrier density at a high AC frequency when the total carrier density tends to saturate with the further increase of the AC frequency. The trap density at a much lower AC frequency (large E_ω) can be estimated by subtracting the estimated free carrier density from the total carrier density measured at the low AC frequency. The specific parameters were determined according to previous reports.[4,5]

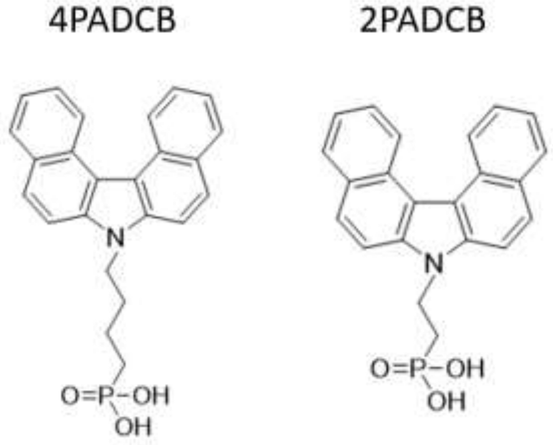

Fig. S1 Chemical Structures of 4PADCB and 2PADCB.

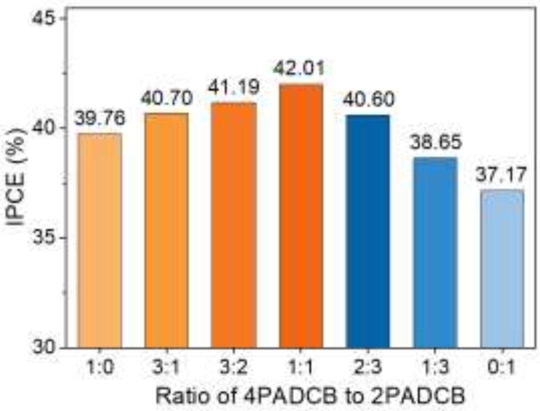

Fig. S2 The IPCE distribution of the PIPVs with different mixing ratio of 4PADCB and 2PADCB.

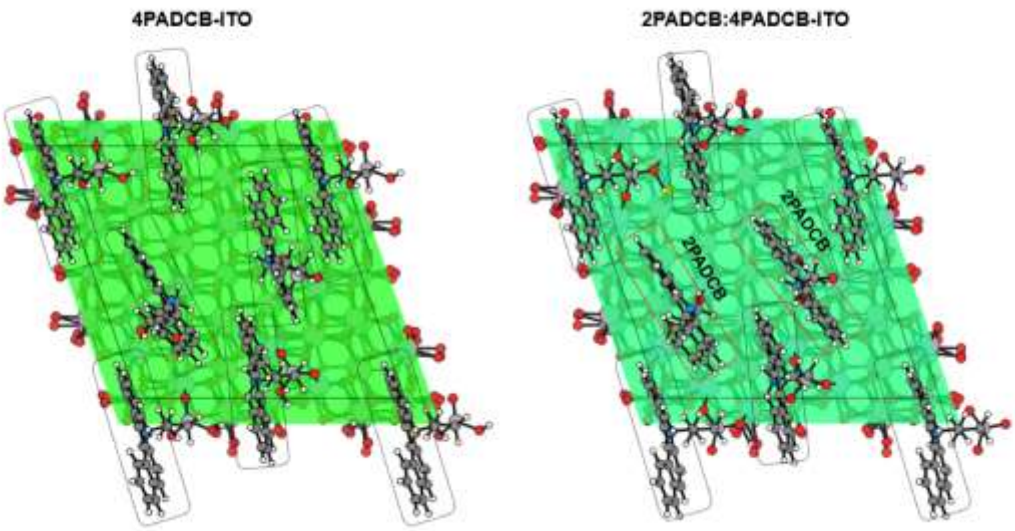

Fig. S3 The top-view image of theoretical SAM morphology on ITO.

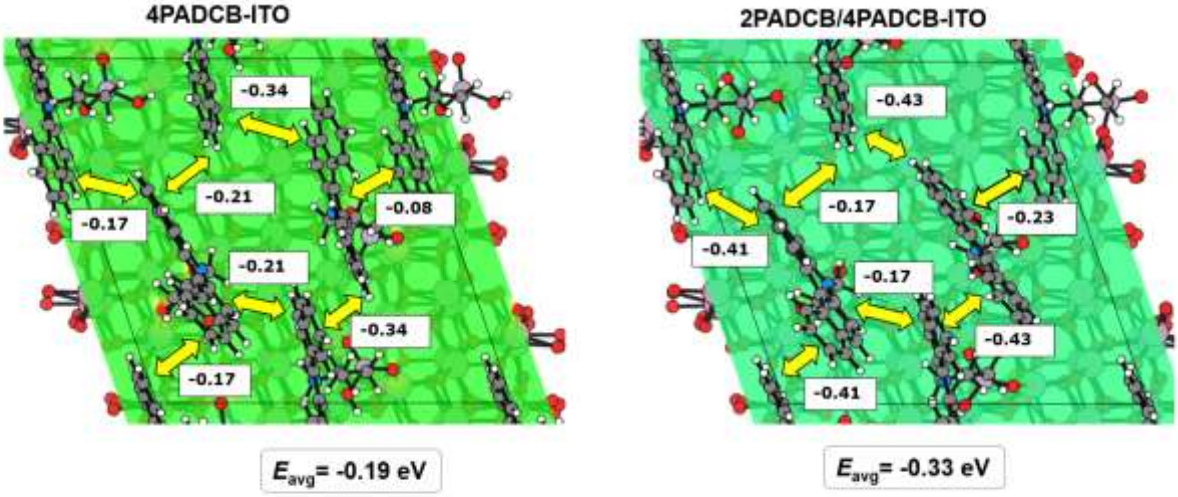

Fig. S4 Theoretical models of the interaction between SAMs.

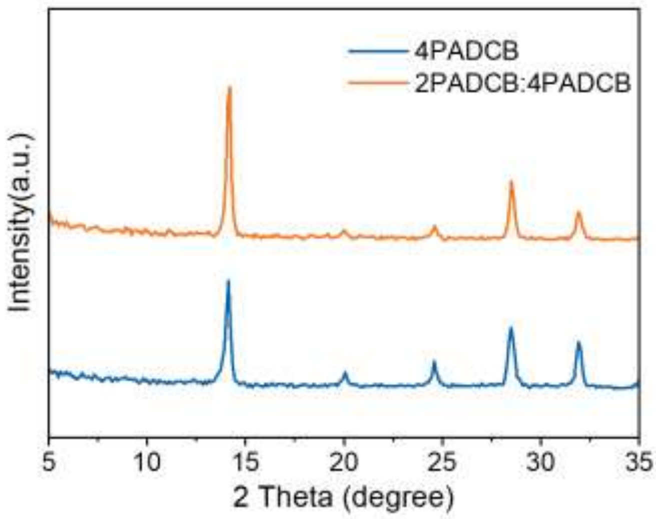

Fig. S5 The XRD curves for the ITO/4PADCB/perovskite and ITO/2PADCB:4PADCB/perovskite films.

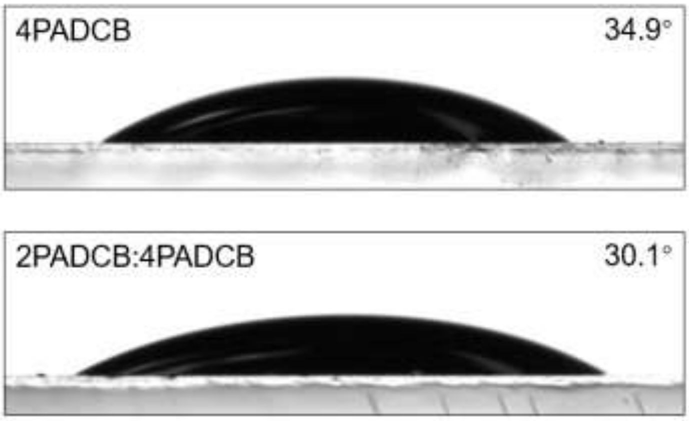

Fig. S6 Contact angles of the perovskite precursor solution on different SAMs.

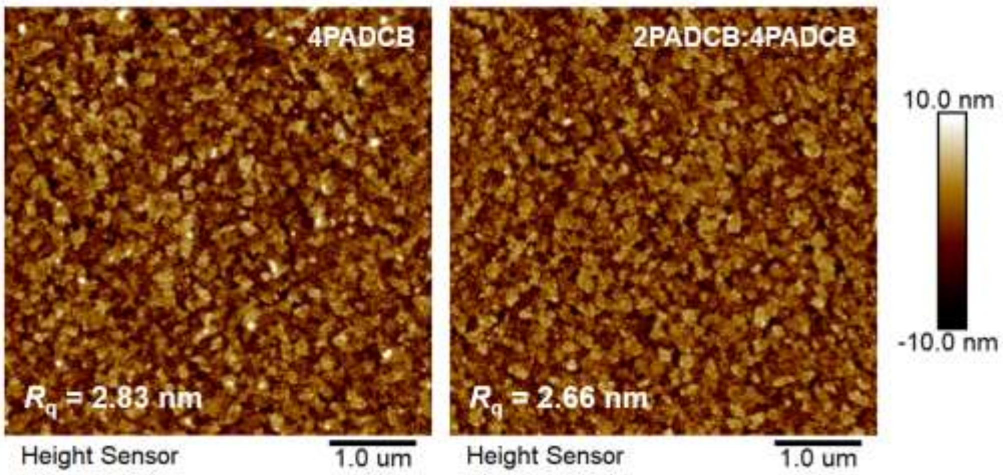

Fig. S7 AFM images of the SAM layers on ITO.

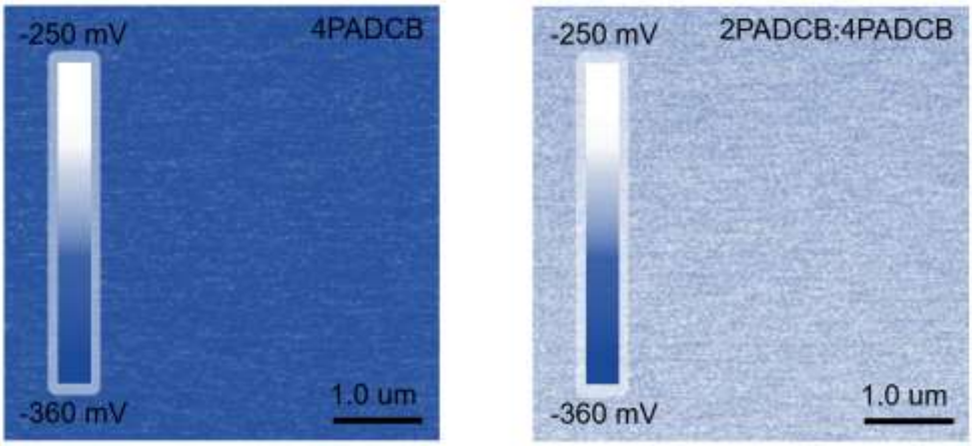

Fig. S8 KPFM images of the 4PADCB and 2PADCB:4PADCB SAM films.

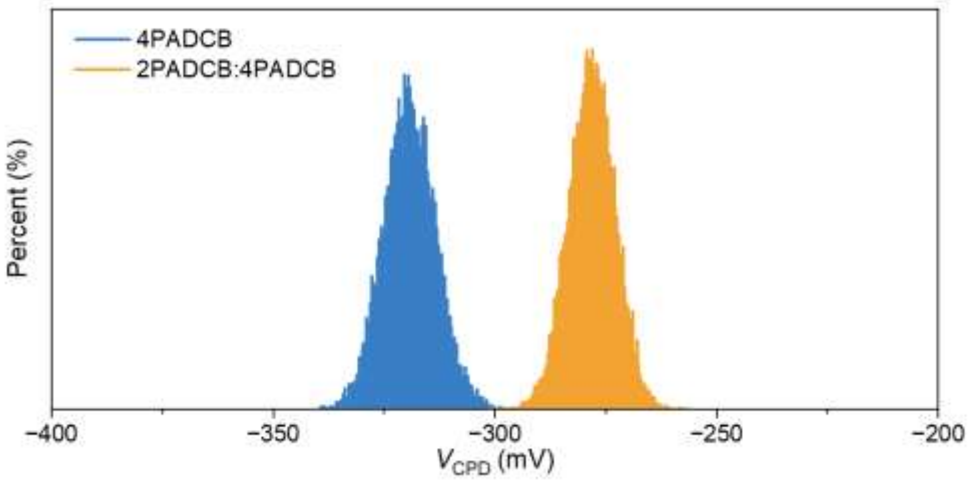

Fig. S9 CPD results of the 4PADCB and 2PADCB:4PADCB SAM films.

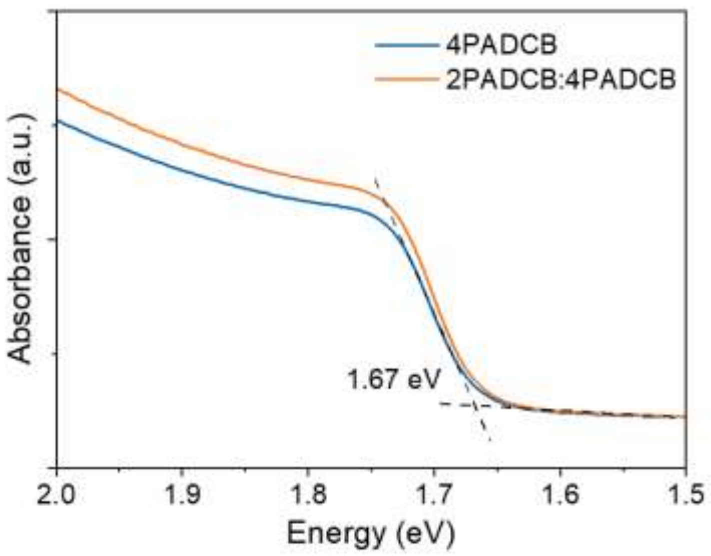

Fig. S10 The UV-vis absorption spectra of the perovskite films on pristine and target SAMs.

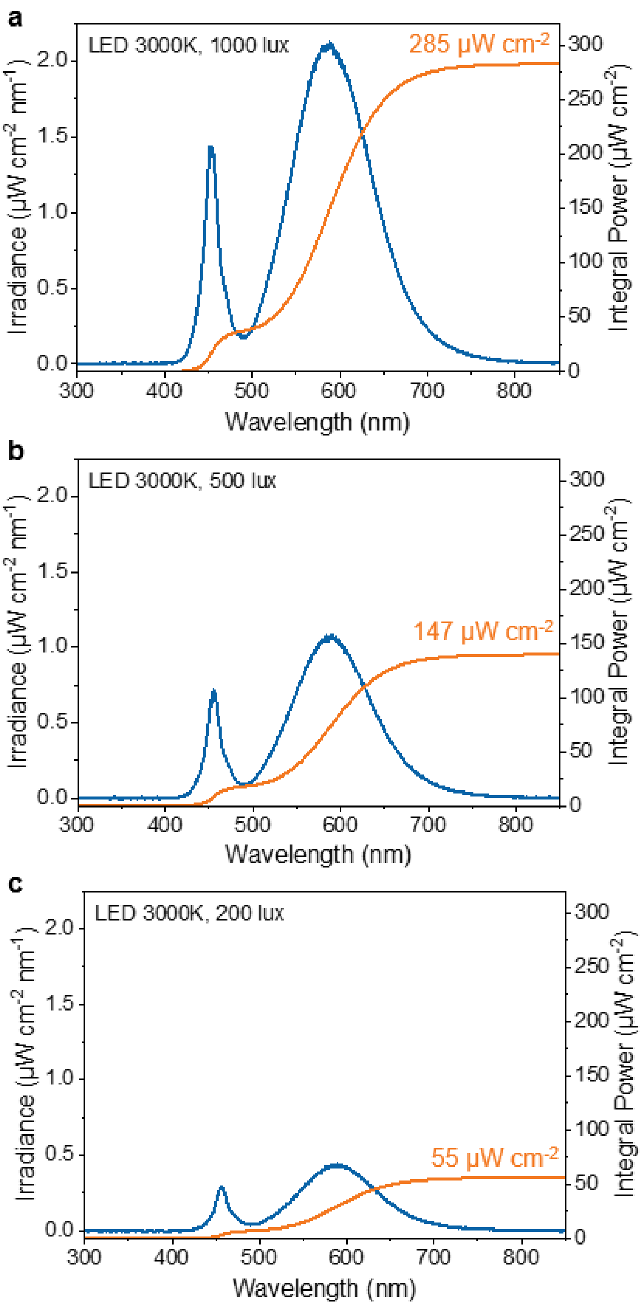

Fig. S11 The spectrum of LED 3000 K light source under **(a)** 1000, **(b)** 500, and **(c)** 200 lux illuminance and the corresponding integrated light source power.

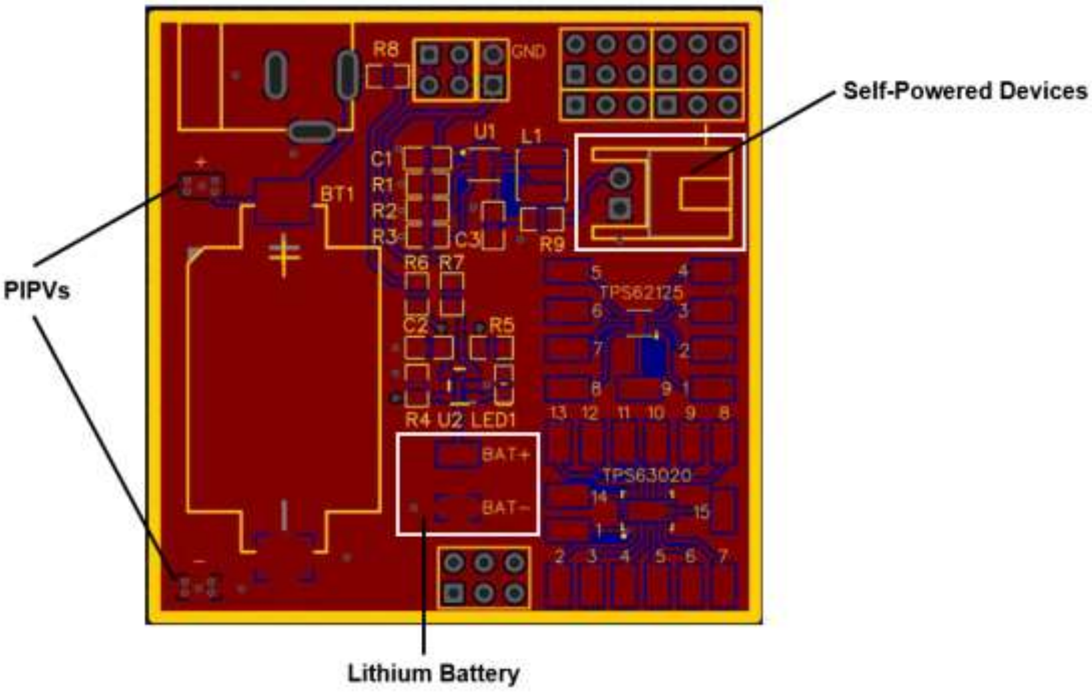

Fig. S12 The integrated energy storage circuit board that combines PIPV panels, lithium batteries, and self-powered devices.

Table S1 The parameters of three kinds of PIPVs under 1000 lux.

Device	Scan Mode	Illumination	J_{SC} (mA/cm^2)	V_{OC} (V)	FF (%)	IPCE (%)	P_{in} (μW/cm^2)	P_{out} (μW/cm^2)
4PADCB	Forward	1000	0.130	1.03	77.65	37.14	280	104
4PADCB	Reverse	1000	0.131	1.05	81.38	39.76	280	111
2PADCB:4PADCB	Forward	1000	0.130	1.06	81.78	40.22	280	113
2PADCB:4PADCB	Reverse	1000	0.131	1.07	84.22	42.01	280	118
2PADCB:4PADCB (1.00 cm^2)	Forward	1000	0.129	0.85	84.57	33.20	280	93
2PADCB:4PADCB (1.00 cm^2)	Reverse	1000	0.128	0.90	82.17	33.87	280	95

Table S2 The parameters of mixed SAM-based PIPVs under different illumination (200, 500, and 1000 lux).

**Device**	**Scan Mode**	**Illumination**	**J_{SC} (mA/cm^2)**	**V_{OC} (V)**	**FF (%)**	**IPCE (%)**	**P_{in} (μW/cm^2)**	**P_{out} (μW/cm^2)**
2PADCB:4PADCB	Forward	200	0.0260	0.99	75.53	34.72	55	19
	Reverse	200	0.0257	1.01	81.57	38.55	55	21
	Forward	500	0.0643	1.04	79.63	37.91	147	53
	Reverse	500	0.0647	1.05	82.10	39.53	147	56
	Forward	1000	0.132	1.07	81.02	40.06	285	114
	Reverse	1000	0.132	1.07	82.80	41.25	285	118

Table S3 The parameters of IPCE and V_{OC}*FF of the published PIPV works in recent years.

Time	V_{OC} (V)	FF (%)	V_{OC}*FF (V)	E_g (eV)	IPCE (%)	Ref.
2015.10	0.85	77	0.654	1.60	27.40	[6]
2018.07	0.87	75.2	0.654	1.77	35.20	[7]
2019.12	0.93	60	0.558	1.61	34.86	[8]
2019.08	1.03	76.8	0.789	1.80	36.20	[9]
2020.12	1.08	83	0.896	1.75	35.60	[10]
2020.02	0.99	80	0.792	1.70	34.00	[11]
2020.09	0.82	68.8	0.564	1.66	34.50	[12]
2020.09	0.75	62	0.465	1.88	28.48	[13]
2021.10	0.93	75.7	0.704	1.57	35.70	[14]
2021.12	0.93	82.1	0.763	1.60	39.20	[15]
2021.04	0.85	53	0.450	1.59	40.24	[16]
2021.05	1.00	79.52	0.795	1.59	40.10	[17]
2021.08	0.89	81	0.720	1.61	32.90	[18]
2021.09	0.96	78.7	0.755	1.60	38.20	[19]
2022.10	1.02	79.7	0.812	1.76	32.61	[20]
2022.02	0.96	79	0.758	1.57	37.90	[21]
2022.02	0.92	77.1	0.709	1.55	31.85	[22]
2022.08	1.15	82	0.943	1.91	33.68	[23]
2023.06	1.07	83	0.888	1.70	41.20	[24]
2023.09	0.94	75.88	0.710	1.63	35.99	[25]
2023.09	1.07	79.2	0.847	1.62	39.90	[26]
2024.03	0.98	84.32	0.825	1.53	41.33	[27]
2024.05	0.99	77.4	0.767	1.75	41.10	[28]
2025.01	1.07	84.22	0.901	1.67	42.01	This work

References

1. Kresse, G., Hafner, J., *Ab initio* molecular dynamics for liquid metals. *Phys. Rev. B* 47, 558 (1993).

2. Kresse, G., Furthmüller, J., Efficiency of *ab-initio* total energy calculations for metals and semiconductors using a plane-wave basis set. *Comput. Mater. Sci.* 6, 15 (1996).

3. Heath, J. T., Cohen, J. D. and Shafarman, W. N., Bulk and metastable defects in $CuIn_{1-x}Ga_xSe_2$ thin films using drive-level capacitance profiling. *J. Appl. Phys.* 95, 1000-1010 (2004).

4. Bleicher, M. and Lange, E., Schottky-barrier capacitance measurements for deep level impurity determination. *Solid-State Electron.* 16, 375-380 (1973).

5. Ni, Z. *et al.* Resolving spatial and energetic distributions of trap states in metal halide perovskite solar cells. *Science* 367, 1352-1358 (2020).

6. Chen, C. Y. *et al.* Perovskite photovoltaics for dim-light applications. *Adv. Funct. Mater.* 25, 7064-7070 (2015).

7. Li, M. *et al.* Interface modification by ionic liquid: a promising candidate for indoor light harvesting and stability improvement of planar perovskite solar cells. *Adv. Energy. Mater.* 8, 1801509 (2018).

8. Dong, C. *et al.* Lead Oxalate-Induced Nucleation Retardation for High-Performance Indoor and Outdoor Perovskite Photovoltaics. *ACS Appl. Mater. Interfaces* 12, 836-843 (2019).

9. Cheng, R. *et al.* Tailoring triple-anion perovskite material for indoor light harvesting with restrained halide segregation and record high efficiency beyond 36%. *Adv. Energy. Mater.* 9, 1901980 (2019).

10. Li, Z. *et al.* Minimized surface deficiency on wide-bandgap perovskite for efficient indoor photovoltaics. *Nano Energy* 78, 105377 (2020).

11. Sun, H. *et al.* Realizing stable artificial photon energy harvesting based on perovskite solar cells for diverse applications. *Small* 16, 1906681 (2020).

12. Lim, J. W. *et al.* Unprecedentedly high indoor performance (efficiency> 34%) of perovskite photovoltaics with controlled bromine doping. *Nano Energy* 75, 104984 (2020).

13. Wang, K. L., Li, X. M., Lou, Y. H., Li, M. and Wang, Z. K., $CsPbBrI_2$ perovskites with low energy loss for high-performance indoor and outdoor photovoltaics. *Sci. Bull.* 66, 347-353 (2021).

14. Xu, J. *et al.* Impermeable inorganic "walls" sandwiching perovskite layer toward inverted and indoor photovoltaic devices. *Nano Energy* 88, 106286 (2021).

15. Li, N. *et al.* Engineering the hole extraction interface enables single-crystal $MAPbI_3$ perovskite solar cells with efficiency exceeding 22% and superior indoor response. *Adv. Energy. Mater.* 12, 2103241 (2022).

16. Dong, C. *et al.* Lycopene-based bionic membrane for stable perovskite photovoltaics. *Adv. Funct. Mater.* 31, 2011242 (2021).

17. He, X. *et al.* 40.1% record low-light solar-cell efficiency by holistic trap-passivation using micrometer-thick perovskite film. *Adv. Mater.* 33, 2100770 (2021).

18. Saranin, D. *et al.* Hysteresis-free perovskite solar cells with compact and nanoparticle NiO for indoor application. *Sol. Energy Mater. Sol. Cells* 227, 111095 (2021).

19. Opoku, H. *et al.* A tailored graft-type polymer as a dopant-free hole transport material in indoor perovskite photovoltaics. *J. Mater. Chem. A* 9, 15294-15300 (2021).

20. Zhang, C. *et al.* Br vacancy defects healed perovskite indoor photovoltaic modules with certified power conversion efficiency exceeding 36%. *Adv. Sci.* 9, 2204138 (2022).

21. Yang, F. *et al.* Enhancement of exciton separation in indoor perovskite photovoltaics by employing conjugated organic chromophores. *J. Power Sources* 520, 230785 (2022).

22. Chen, C. H. *et al.* Full-dimensional grain boundary stress release for flexible perovskite indoor photovoltaics. *Adv. Mater.* 34, 2200320 (2022).

23. Jiang, S. *et al.* Efficient Perovskite Indoor Photovoltaics with Open-Circuit Voltage of 1.15 V via Collaborative Optimization of $CsPbI_2Br$ Layer and Hole Transport Layer. *Small Methods* 6, 2200624 (2022).

24. Wang, K. L. *et al.* Ion-dipole interaction enabling highly efficient $CsPbI_3$ perovskite indoor photovoltaics. *Adv. Mater.* 35, 2210106 (2023).

25. Han, E. Q. *et al.* High-Performance Indoor Perovskite Solar Cells by Self-Suppression of Intrinsic Defects via a Facile Solvent-Engineering Strategy. *Small* 20, 2305192 (2024).

26. Jiang, B. H. *et al.* Enhancing the Efficiency of Indoor Perovskite Solar Cells through Surface Defect Passivation with Coplanar Heteroacene Cored A-D-A-type Molecules. *Adv. Funct. Mater.* 34, 2312819 (2024).

27. Liu, C. *et al.* Flexible Indoor Perovskite Solar Cells by In Situ Bottom-Up Crystallization Modulation and Interfacial Passivation. *Adv. Mater.* 36, 2311562 (2024).

28. Kim, S. J. *et al.* Ultrahigh-performance indoor perovskite quantum dot photovoltaics via ligand-passivation engineering. *Chem. Eng. J.* 488, 151154 (2024).
